# Transcatheter Left Ventricular Restoration in Ischemic Heart Failure and Dilated Cardiomyopathy

**DOI:** 10.1002/ccd.70425

**Published:** 2025-12-15

**Authors:** Muhammad Mohid Haroon, Faizan Ahmad, Ramsha Ali, Saqlain Haider, Areehah Zafar Masood, Usama Yaseen, Taha Ahmad Zaka Ur Rehman, Muhammad Abdullah Sardar, Muhammad Umer, Shruthi Aswathappa, Muhammad Salman Sabri, Muhammad Muneeb Murtaza, Rubiya Ali, Sunny Kumar, Sameer Ali, Najam Gohar, Mohamed Bakr, Swapnil Patel, Mohammad A. Hussain, Fawaz Alenezi

**Affiliations:** ^1^ Ameer‐ud‐Din Medical College/Lahore General Hospital Lahore Pakistan; ^2^ Department of Medicine, Jersey Shore University Medical Centre Hackensack Meridian Health Neptune New Jersey USA; ^3^ Peoples University of Medical and Health Sciences Nawabshah Pakistan; ^4^ Department of Medicine Ziauddin Medical College Karachi Pakistan; ^5^ Internal Medicine University of Oklahoma Health Sciences Center Oklahoma City Oklahoma USA; ^6^ King Edward Medical University Lahore Pakistan; ^7^ M S Ramaiah Medical College Bengaluru India; ^8^ Jefferson Abington Hospital Thomas Jefferson University Philadelphia Pennsylvania USA; ^9^ Memorial Healthcare System Hollywood Florida USA; ^10^ Wright Center for Graduate Medical Education Scranton Pennsylvania USA; ^11^ Desert Valley Hospital Victorville California USA; ^12^ Division of Cardiology, Department of Medicine Duke University School of Medicine Durham North Carolina USA

**Keywords:** dilated cardiomyopathy, Ischemic heart failure, left ventricular remodeling, minimally invasive therapy, transcatheter left ventricular restoration, ventricular function

## Abstract

Ischemic heart disease is the leading global cause of death and frequently progresses to ischemic heart failure (IHF) after myocardial infarction through adverse left ventricular (LV)remodeling. Surgical LV restoration improves ventricular geometry and function but is limited by invasiveness and inconsistent outcomes. Transcatheter Left Ventricular Restoration (TLVR) has emerged as a minimally invasive alternative,employing devices such as Revivent TC, AccuCinch, and Parachute to reduce LV volume, wall stress, and improve LV ejection fraction (LVEF). While several studies have reported promising results, no prior meta‐analysis has synthesized the evidence across devices. This meta‐analysis followed PRISMA 2020 guidelines. PubMed, Embase, Scopus, and Cochrane Library were searched through July 2025. Eligible studies included adult patients with IHF or dilated cardiomyopathy undergoing TLVR and reporting outcomes including LVEF, LV end‐diastolic volume (LVEDV), New York Heart Association (NYHA) class, or Kansas City Cardiomyopathy Questionnaire (KCCQ). Single‐arm interventional and observational cohort studies were included. Data extraction was performed by four reviewers, with bias assessed using ROBINS‐I and the Newcastle–Ottawa Scale. Pooled mean differences (MD) were calculated using a random‐effects model (Hartung–Knapp–Sidik–Jonkman). Seventeen studies were included, with baseline LVEF 22.8%–38% and LVEDV 75–235 mL. TLVR significantly reduced LVEDV (MD −25.94 mL; *p* < 0.00001), increased LVEF (+6.69%; *p* < 0.00001), and improved the NYHA class (MD −0.73; *p* = 0.02). KCCQ scores improved in some studies but were not significant overall. Revivent TC and AccuCinch showed consistent benefits, whereas Parachute outcomes were more variable. This meta‐analysis of TLVR demonstrates consistent improvements in LV remodeling, function, and symptoms. Nonetheless, high heterogeneity, small cohorts, and limited follow‐up highlight the need for large randomized trials to establish durability, survival benefit, and refine patient selection.

## 1 Introduction

Ischemic heart disease (IHD) is the leading cause of death worldwide and, if not treated or diagnosed, can develop into ischemic heart failure (IHF) [1]. Following acute myocardial infarction (AMI), adverse left ventricular (LV) remodeling often occurs, with increased LV volumes and reduced ejection fraction (LVEF) due to increased wall tension, loss of orientation of the myocardial fibers, and scarring.

Evidence shows that both pharmacologic and non‐pharmacologic therapies such as cardiac resynchronization therapy can improve survival in patients with LV systolic dysfunction by reducing LV volumes, either ESV or EDV, with bigger reductions associated with better survival outcomes [2]. Where medical treatment is insufficient, surgical interventions are available to correct the form, size, and function of the LV [3].

Surgical restoration of the LV has been used to remodel the dilated failing ventricle and improve chamber architecture and performance. Its use is limited, however, by the invasiveness of the procedure and heterogeneity of outcomes across studies [4].

Transcatheter Left Ventricular Restoration (TLVR) is an alternative, less invasive method attempting to reduce LV volume, wall stress, and improve EF by plicating fibrotic scar tissue [1]. AccuCinch system, BioVentrix LIVE system, Parachute device, and Revivent TC system are examples of the systems investigated in this patient population.

While individual studies have reported promising improvement in EF and quality of life following TLVR, no meta‐analysis to our knowledge has synthesized the evidence collectively or compared device results. This study aims to address this gap by systematically reviewing the literature on TLVR and performing a comparative analysis of device‐specific outcomes.

## 2 Methods

### 2.1 Study Design and Protocol Registration

This systematic review and meta‐analysis was conducted in line with the updated 2020 guidelines of the Preferred Reporting Items for Systematic Reviews and Meta‐Analyses (PRISMA). The review protocol was prospectively registered in PROSPERO (Registration ID: CRD420251231767). The protocol was developed a priori following the PICO framework to ensure methodological rigor and transparency. The review protocol was developed a priori in accordance with the PICO framework.

### 2.2 Eligibility Criteria

Studies were included if they met the following criteria:

#### 2.2.1 Population (P)

Adult patients with ischemic heart failure (IHF) or dilated cardiomyopathy undergoing transcatheter left ventricular restoration (TLVR).

#### 2.2.2 Intervention (I)

TLVR performed using devices such as Revivent TC, AccuCinch, Heartech LV Partitioning Device, Parachute device, or similar technologies.

#### 2.2.3 Comparator (C)

Only single‐arm designs were included, and no direct comparator arm was required. This restriction was applied because comparative data for TLVR remain limited, and most published studies report single‐arm outcomes.

#### 2.2.4 Outcomes (O)

At least one of the following outcomes reported: left ventricular ejection fraction (LVEF), left ventricular end‐diastolic volume (LVEDV), New York Heart Association (NYHA) functional class, Kansas City Cardiomyopathy Questionnaire (KCCQ) scores.

We included prospective and retrospective observational studies, non‐randomized clinical trials, and randomized controlled trials reporting single‐arm TLVR outcomes.

#### 2.2.5 Exclusion Criteria

Case reports, review articles, editorials, conference abstracts without full data, animal studies, and studies without extractable outcome measures were excluded.

### 2.3 Search Strategy and Databases

A comprehensive literature search was conducted in PubMed/MEDLINE, Embase, Scopus, and the Cochrane Library covering studies from inception to July 2025. The search strategy combined controlled vocabulary (e.g., MeSH terms) and relevant keywords, including “transcatheter left ventricular restoration,” “Parachute device,” “Revivent TC,” “AccuCinch,” and “ventricular partitioning.” Boolean operators AND and OR were used to combine search terms.

All retrieved articles were imported into Rayyan AI for initial screening. Title and abstract screening was performed first, followed by removal of duplicates within Rayyan. Four reviewers independently performed full‐text screening of potentially eligible studies, with disagreements resolved through consensus or, when necessary, adjudicated by a senior reviewer.

### 2.4 Study Selection

**Figure 1:**
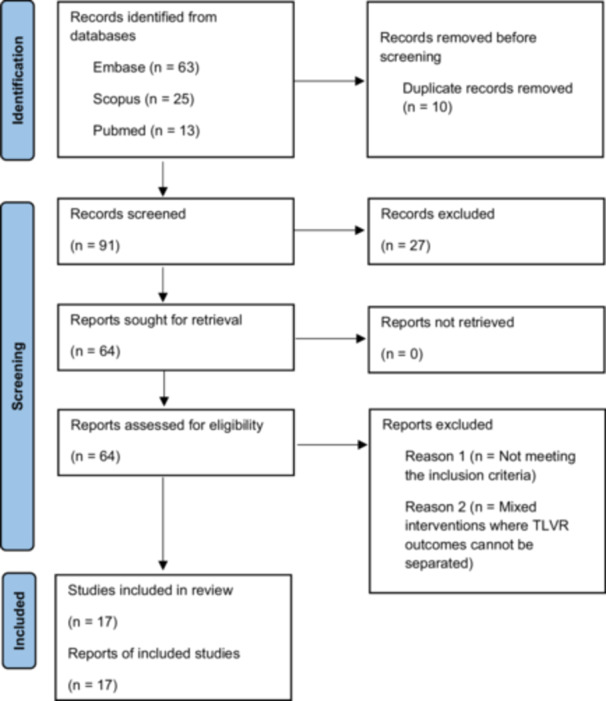
PRISMA flow diagram for transcatheter left ventricular restoration systematic review. [Color figure can be viewed at wileyonlinelibrary.com]

A total of 101 records were retrieved from electronic databases. Ten duplicate records were removed within Rayyan AI, leaving 91 records for title and abstract screening by six independent reviewers. During this stage, 27 records were excluded, and the remaining 63 reports were sought for retrieval. Two reports could not be retrieved, resulting in 61 full‐text articles assessed for eligibility. Four reviewers then conducted detailed full‐text screening, after which 17 studies met the inclusion criteria and were included in the final review. Any disagreements at any stage were resolved through discussion or consultation with a senior reviewer (PRISMA flow diagram, Figure 1).

### 2.5 Data Extraction

Four independent reviewers used a standardized data extraction sheet in Microsoft Excel to document all relevant information. The following data were collected:

Study profile: Study ID, first author, year of publication, country, study design, sample size, follow‐up duration.

### 2.6 Baseline Characteristics

Mean age, percentage of male patients, baseline LVEF, baseline LVEDV, NYHA class distribution, comorbidities, and medication use.

#### 2.6.1 Intervention Details

Device type, procedural technique, and peri‐procedural protocols.

#### 2.6.2 Outcomes

Baseline and follow‐up values for LVEF, LVEDV, LV end‐systolic volume (LVESV), NYHA class, KCCQ scores.

### 2.7 Quality Assessment

The methodological quality of included studies was assessed using an adapted case–control version of the Newcastle–Ottawa Scale (NOS). The NOS evaluates three key domains: Selection, including the adequacy of case definitions, representativeness of cases, selection of controls, and definition of controls; Comparability, assessing whether cases and controls are comparable based on key confounding variables; and Exposure, which examines the ascertainment of exposure, consistency of exposure assessment across groups, and the extent of non‐response. Detailed NOS scoring for all included studies is presented in the Supporting information Table 1.

### 2.8 Statistical Analysis

All statistical analyses were performed using Revman web software. Continuous outcomes were pooled as mean difference (MD) with standard error (SE) were calculated using a random‐effects model. The Hartung–Knapp–Sidik–Jonkman (HKSJ) method was applied to compute 95% confidence intervals (CIs). Between‐study variance (τ²) was estimated using the Restricted Maximum Likelihood (REML) method. Heterogeneity was assessed using the Cochran's Q test (χ²) and quantified with the I² statistic, with *p* < 0.10 indicating significant heterogeneity. A test for the overall effect was conducted using a t‐statistic, and results with *p* < 0.05 were considered statistically significant. Publication bias was evaluated using funnel plot asymmetry and Egger's regression test. Sensitivity analyses were conducted by sequentially excluding each study to assess the robustness of pooled estimates.

## 3 Results

### 3.1 Study Selection

The initial database search identified 101 records. After removing 10 duplicates, 91 unique articles were screened by title and abstract. Of these, 27 were excluded for not meeting the inclusion criteria. The remaining 64 underwent full‐text screening, and 17 studies met the eligibility criteria for inclusion (Figure 1).

### 3.2 Study Characteristics

Seventeen studies (Table 1) evaluating transcatheter left ventricular restoration (TLVR) in patients with heart failure were included in the pooled analysis. Across studies, the study population comprised predominantly older adults (mean age 58–62 years) and males (65%–94%), reflecting the typical TLVR demographic. Baseline left ventricular ejection fraction (LVEF) ranged from 22.8% to 38%, indicating severe systolic dysfunction. Left ventricular end‐diastolic volume (LVEDV) varied widely (75–235 mL), with the largest volumes observed in Parachute device cohorts, reflecting greater ventricular dilation in these populations.

**Table 1:** Baseline characteristics of patients undergoing transcatheter left ventricular restoration (TLVR).

Author name	Year	*n*	Age (mean ± SD)	% Male	LVEF (%)	LVEDV (mL)	NYHA Class (Mean/% III–IV)	KCCQ score	Ischemic HF %	Device used	Follow‐up (months)
Alaiti et al.	2016	32	59.6 ± 8.0	81%	27.6 ± 6.9	122.4 ± 26.9	2.7/100% III–IV	N/A	100%	Parachute®	6
Bozdag‐Turan et al. (Parachute)	2013	8	62 ± 10	63%	32 ± 6	122.2 ± 27.8	2.8 ± 0.7	—	100%	Parachute LVPD	3
Costa et al. (Parachute FIH)	2012	39	N/A	N/A	15–40	127.5	2.0/N/A	N/A	100%	Parachute	36
Costa et al.	2014	39	56.4 ± 9.8	88%	27.0 ± 1.1	127.7 ± 2.9	2.7/100% II–IV	N/A	100%	Parachute®	36
Hamid et al.	2023	51	56.3 ± 13.1	86%	29.2	206	46% III–IV	61.4	26%	AccuCinch TLVR	12
Hegeman et al.	2022	30	62 ± 12	80.00%	< 40	84 ± 32 (index)	3 ± 1/76%	—	100%	Revivent TC System	40.8 (3.4 years)
Klein et al. (ICVTS)	2019	9	60 ± 8	89%	28 ± 8	75 ± 23 (indexed)	2.7 ± 0.4/Not reported	Not reported	100%	Revivent TC	Not reported
Klein et al.	2019	89	60.4 ± 9.9	80%	29 ± 8	106 ± 33 (indexed)	2.6 ± 0.5/59% III	Not reported	100%	Revivent TC	12
Loforte et al.	2019	7	72 ± 8.9	71%	22.8 ± 8.1	137.2 ± 20.1 (indexed)	3.4 ± 0.6/100% III–IV	Not reported	100%	Revivent TC	6.3 (mean)
Mazzaferri et al.	2012	39	56.4 ± 8.6	87%	≤ 40	127.2 ± 4.2 (index)	3/76%	38.6 ± 5.1	100%	Parachute Device	12
Naar et al. (Revivent TC)	2021	23	59 ± 11	65%	32 ± 7	107 ± 27	2.3/13%	N/A	100%	Revivent TC	61.2 (mean)
PARACHUTE III (Thomas et al.)	2015	100	62.8 ± 10.4	81%	29.2 ± 7.9	117.3 ± 26.3 (indexed)	2.6 ± 0.5/56% III	Not reported	100%	Parachute	12
Patterson et al. (Parachute III)	2017	10	61.0 ± 10.4	90%	38 ± 11	235 ± 81	2.6/40%	N/A	100%	Parachute	6
Yang et al.	2016	31	57.1 ± 10.4	94%	30	110.8	6.4% III	—	100%	Parachute® LVPD	3
Yun et al.	2017	28	58.3 ± 7.2	79%	Not reported	Not reported	20 (71.4% III)	Not reported	100%	Parachute LVPD	6
Zhu et al.	2019	16	60.9 ± 9.8	87.50%	32.5	106.3	50% III	—	100%	Heartech® LVPD	1.2 (36 days)
Zhu et al.	2022	16	60.93 ± 9.75	88%	32.75 ± 6.84	105.00 (IQR: 90.00–130.00)	2.7 (71.4% III)	Not reported	100%	Heartech LVPD	12 ± 1

Abbreviations: IQR, interquartile range; KCCQ, kansas city cardiomyopathy questionnaire; LVEDV, left ventricular end‐diastolic volume; LVEF, left ventricular ejection fraction; LVPD, left ventricular partitioning device; *N/A,* not applicable; NR, not reported; NYHA, new york heart association; TLVR, transcatheter left ventricular restoration.

Most patients were New York Heart Association (NYHA) Class II–IV at baseline, with advanced symptoms (Class III–IV) more frequent in Revivent TC studies (59%–76%) than in Parachute trials (e.g., 6.4% Class III in Yang et al.). Ischemic cardiomyopathy was the predominant etiology in nearly all studies except Hamid et al., where 26% of patients had non‐ischemic heart failure. Follow‐up durations ranged from 1.2 months (Zhu et al.) to 5 years (Naar et al.) (Table 1).

### 3.3 LVEDV Outcomes

All studies demonstrated post‐procedural LVEDV reduction, ranging from –76.5 mL to –1.14 mL, with the most pronounced decreases reported by Loforte et al. (–59.2 mL) and Hamid et al. (–33.6 mL). These findings indicate consistent reverse remodeling following TLVR (Table 2).

**Table 2:** Left ventricular end‐diastolic volume (LVEDV) changes pre‐ and post‐intervention.

Study	Year	*n*	Pre‐LVEDV mean (SD)	Post‐LVEDV mean (SD)	Mean difference	95% CI	Weight (%)
Alaiti et al.	2016	32	122.4 (27.1)	104.2 (30.3)	−18.2	−25.1, −11.3	100
Bozdag‐Turan	2013	8	Not reported	Not reported	−76.5	−116.0 to −36.8	100
Costa	2012	31	127.7 (2.92)	105.8 (3.02)	−21.9	[−22.5, −21.3]	30
Costa	2014	39	127.5 ± N/A	106.7 ± N/A	−20.8	N/A	N/A
Hamid et al.	2023	51	206.0 (55.8)	172.4 (52.8)	−33.6	[−44.6, −22.6]	50
Hegeman	2022	30	84 ± 32	58 ± 25	−26	−32 to −20	30
Klein et al. (2018)	2019	9	75.0 (23.0)	45.0 (6.0)	−30	[−45.2, −14.8]	10
Klein et al. (2019)	2019	67	106.0 (33.4)	80.0 (26.0)	−26	[−31.2, −20.8]	25
Loforte et al.	2019	7	137.2 (20.1)	78.0 (10.2)	−59.2	[−75.1, −43.3]	15
Mazzaferri	2012	28	127.2 (4.2)	110.4 (4.6)	−16.8	[14.2, 19.4]	33.3
Naar	2021	23	107 ± 27	82 ± 21	−25	−31 to −19	19
Patterson	2015	10	235 (81)	100 ± 55	−22	−34 to −9	20
Thomas et al.	2017	64	117.3 (26.3)	99.1 (27.3)	−18.2	[−22.4, −14.0]	20
Yang et al.	2016	31	110.8 (26.1)	82.1 (21.3)	−28.7	[−37.8, −19.6]	30
Yun et al.	2017	28	97.66 (34.68)	96.52 (33.04)	−1.14	[−6.00, 3.72]	33.3
Zhu et al.	2019	16	105.00 (90.00–130.00)*	76.50 (57.75–120.25)*	−28.5	[−40.00, −17.00]	33.3
Zhu et al.	2022	16	106.27 (28.01)	83.20 (16.87)	−23.07	[−36.27, −9.87]	20

Abbreviations: CI, confidence interval; LVEDV, left ventricular end‐diastolic volume; Median (IQR); N/A, not available; SD, standard deviation.

### 3.4 LVEF Outcomes

Baseline LVEF ranged from 22.8% to 38%. Post‐intervention improvements varied between +1.8% (Thomas et al., 2017) and +14% (Naar et al., 2021), with the largest relative gain observed in Klein et al. Notably, larger trials such as Hamid et al. (*n* = 51) and Thomas et al. (*n* = 97) also demonstrated clinically meaningful improvements (Table 3).

**Table 3:** Left ventricular ejection fraction (LVEF) changes pre‐ and post‐intervention.

Study	Year	Sample size (*n*)	Pre‐LVEF mean (SD)	Post‐LVEF mean (SD)	Mean difference	95% CI	Weight (%)
Alaiti et al.	2016	32	27.5 (6.9)	31.9 (8.4)	4.4	[2.1, 6.7]	30
Bozdag‐Turan et al.	2013	8	32 ± 6	38.87 ± 6.39*	6.87	(5.36; 8.39)	—
Costa et al. (2012)	2012	39	Not reported	Not reported	Not reported	Not reported	—
Costa et al. (2014)	2014	31	27.0 (1.13)	30.0 (1.17)	3	[NR]	NR
Hamid et al. (AccuCinch)	2023	51	29.2 (4.8)	32.8 (7.3)	3.6	[1.8, 5.4]	63.8
Hegeman et al. (2022)	2022	30	33 ± 8	44 ± 10	11	[9.2, 12.8]	100
Klein et al. (Revivent TC)	2019	64	29 (8)	34 (9)	5	[NR]	NR
Klein et al.	2019	9	28.00 (8.00)	40.00 (10.00)	12	[6.00, 18.00]	100
Loforte et al.	2019	7	22.8 (8.1)	35.0 (7.2)	12.2	[6.8, 17.6]	15
Mazzaferri et al. (2012)	2012	28	26.9 (1.4)	29.4 (1.4)	2.5	[NR]	NR
Naar et al. (2021)	2021	23	32 ± 7	46 ± 15*	14	[10.2, 17.8]	100
Patterson et al. (2017)	2015	10	38 ± 11	46 ± 14	8	[4.1, 11.9]	100
Thomas et al. (PARACHUTE III)	2017	97	29.2 (7.9)	31.0 (7.6)	1.8	[NR]	NR
Yang et al. (2016)	2016	31	30.0 ± 5.4	35.8 ± 6.8	5.8	[3.5, 8.1]	100
Yun et al.	2017	28	31.00 (10.00)	35.00 (10.00)	4	[1.20, 6.80]	100
Zhu et al.	2019	16	32.75 (6.84)	42.50 (IQR: 34.75–50.25)*	9.75	[5.25, 14.25]	100
Zhu et al. (Heartech®)	2022	16	32.47 (6.98)	40.41 (6.15)	7.94	[5.32, 10.56]	20

*Note:* Where marked with asterisk, IQR reported instead of SD.

Abbreviations: 95% CI, 95% confidence interval; IQR, interquartile range*; LVEF, left ventricular ejection fraction (%); NR, not reported; SD, standard deviation.

### 3.5 NYHA Functional Class

Functional capacity improved in all studies, with marked reductions in the prevalence of advanced heart failure. For example, in Yun et al., Class III/IV patients decreased from 71% to 18%, and in Zhu et al., from 50% to 0%. Costa et al. (2012) reported worsening in 15% of patients (Table 4).

**Table 4:** Changes in NYHA functional class following intervention.

Study (Author)	Year	*n*	Pre‐NYHA (mean ± SD or % class III–IV)	Post‐NYHA	Mean/proportional change	95% CI	Weight (%)
Alaiti et al.	2016	32	50% class III	45% class I, 55% class II (improved group)	85% improved/maintained	N/A	N/A
Bozdag‐Turan et al.	2013	8	2.8 ± 0.7 (100% class II–IV)	1.6 ± 0.5	−1.2	−1.7 to −0.7	20
Costa et al.	2012	31	100% class II–IV	52% improved, 33% no change, 15% worsened	48% improvement in LVEF	N/A	N/A
Costa et al.	2014	39	2.0 (NYHA class II–IV)	1.9 (87% symptom improvement)	−0.1 (mean reduction)	N/A	N/A
Hamid et al. (AccuCinch®)	2023	51	46% class III–IV	29% class III–IV	17% reduction in class III–IV	—	—
Hegeman et al.	2022	30	3 ± 1 (76% class III–IV)	1.9 ± 0.6 (76% class I–II)	−1.1 (mean reduction)	N/A	N/A
Klein (Revivent TC)	2019	77	2.6 ± 0.5	1.9 ± 0.8	26% Change	—	—
Klein et al.	2019	9	2.7 ± 0.4 (100% class II–IV)	2.3 ± 0.7	−0.4	−0.9 to +0.1	15
Loforte et al.	2019	7	3.4 ± 0.6	1.4 ± 0.9	2.0 point reduction	N/A	N/A
Mazzaferri et al. (2012)	2012	28	2.5 ± 0.6 (median class III)	1.3 ± 0.6	Mean reduction: −1.2 classes	*p* < 0.001 (no CI provided)	N/A
Naar et al.	2021	23	2.3 ± 0.5	1.6 ± 0.7	−0.7 (mean reduction)	N/A	N/A
Patterson et al.	2015	10	2.5 ± 0.5 (40% class III)	1.9 ± 0.6 (50% class I–II)	−0.6 (mean reduction)	N/A	N/A
Thomas (PARACHUTE III)	2017	97	56% class III, 44% class II	19.6% class I, 46.4% class II, 18.6% class III	43% improved ≥ 1 class	—	—
Yang et al.	2016	31	2.3 ± 0.5 (6.4% class III)	1.6 ± 0.7 (60% class I)	−0.7 (mean reduction)	N/A	N/A
Yun et al. (2017)	2017	28	71.4% class III	17.9% class III	53.5% reduction in class III	N/A	N/A
Zhu et al.	2019	16	2.7 ± 0.4 (100% class II–IV)	1.6 ± 0.5	−1.1	−1.4 to −0.8	25
Zhu et al. (Heartech®)	2022	16	50% class III	0% class III	50% reduction in class III	—	—

*Note*: NYHA class I (mild) to IV (severe); improvements reflect reduced class severity or symptom burden.

### 3.6 KCCQ Scores

Kansas City Cardiomyopathy Questionnaire (KCCQ) scores improved in most studies, reflecting enhanced quality of life and symptom relief. Significant mean increases were reported by Hamid et al. (+16.4) and Zhu et al. (+12.7 to +16.6), whereas Klein et al. and Mazzaferri et al. observed declines. Incomplete paired KCCQ data precluded formal meta‐analysis (Table 5). Overall pooled KCCQ results were not statistically significant (MD + 4.68; 95% CI –13.64 to 22.99; *p* = 0.52), likely due to incomplete reporting and heterogeneity.

**Table 5:** Changes in Kansas City Cardiomyopathy Questionnaire (KCCQ) scores following intervention.

Study	Year	*n*	Pre‐KCCQ mean (SD)	Post‐KCCQ mean (SD)	Mean difference	95% CI	Weight (%)
Alaiti et al.	2016	32	—	—	—	—	—
Bozdag‐Turan et al.	2013	8	—	—	—	—	—
Costa	2012	39	Not reported	Not reported	Not reported	N/A	N/A
Costa et al.	2014	31	Not reported	Not reported	Not reported	N/A	N/A
Hamid et al.	2023	47	61.4 (26.0)	77.7 (19.0)	16.4 (18.7)	[10.9, 22.0]	—
Hegeman et al.	2022	30	Not reported	Not reported	Not reported	N/A	N/A
Klein	2019	9	Not reported	Not reported	Not reported	—	—
Klein	2019	46	39 ± 21	26 ± 22	−13	—	—
Loforte	2019	7	Not reported	Not reported	Not reported	N/A	N/A
Mazzaferri et al.	2012	28	38.6 (6.1)	28.4 (4.4)	−10.2	N/A	N/A
Naar et al.	2021	23	Not reported	Not reported	Not reported	—	—
Patterson et al.	2015	10	Not reported	Not reported	Not reported	—	—
Thomas	2017	100	Not reported	Not reported	Not reported	—	—
Yang et al.	2016	31	Not reported	Not reported	Not reported	—	—
Yun et al.	2017	28	—	—	—	—	—
Zhu et al.	2019	15	65.93 (11.25)	78.67 (8.35)	12.74	[10.9, 22.0]	—
Zhu et al.	2022	16	65.93 (11.25)	82.50 (5.44)	16.57	[12.81, 20.33]	—

Abbreviations: “—“, Not reported/applicable; Negative values indicate worsened scores; CI, confidence interval; KCCQ, Kansas City Cardiomyopathy Questionnaire; SD, standard deviation.

### 3.7 Pooled Outcomes

**Figure 2:**
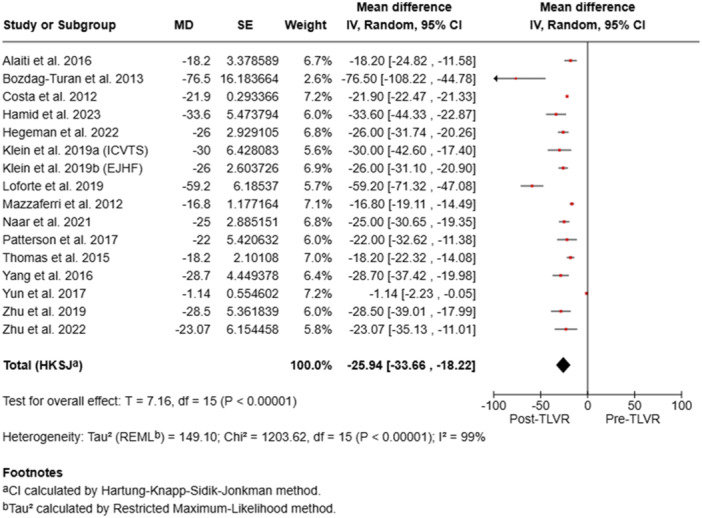
Pooled analysis of studies. [Color figure can be viewed at wileyonlinelibrary.com]

Pooled analyses demonstrated statistically significant improvements in LVEDV (mean difference [MD] –25.94 mL; 95% confidence interval [CI] –33.66 to –18.22; *p* < 0.00001), LVEF (MD + 6.69%; 95% CI 4.68–8.70; *p* < 0.00001), and NYHA class (MD –0.73; 95% CI –1.29 to –0.18; *p* = 0.02), with no significant change in KCCQ scores (MD + 4.68; 95% CI –13.64 to 22.99; *p* = 0.52). Substantial heterogeneity (I² > 90%) was observed across all analyses (Figure 2). Overall, TLVR was associated with favorable ventricular remodeling, improved systolic performance, and better functional capacity in patients with ischemic heart failure, despite considerable between‐study variability.

**Figure 3:**
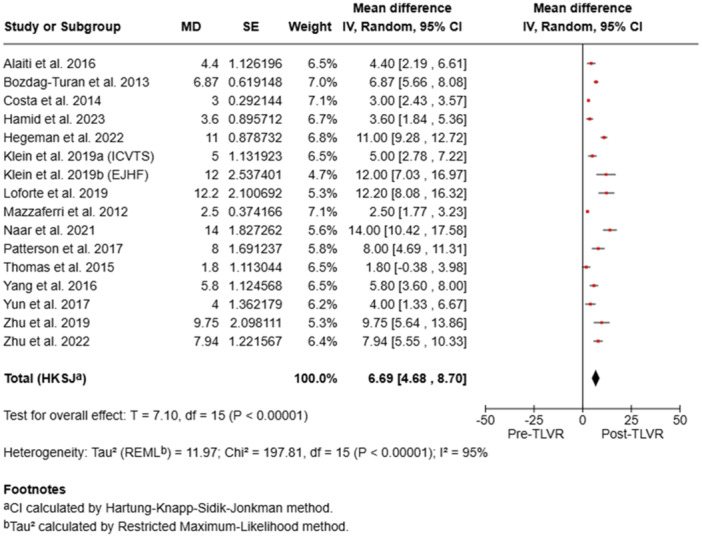
Pooled analysis of studies. [Color figure can be viewed at wileyonlinelibrary.com]

**Figure 4:**
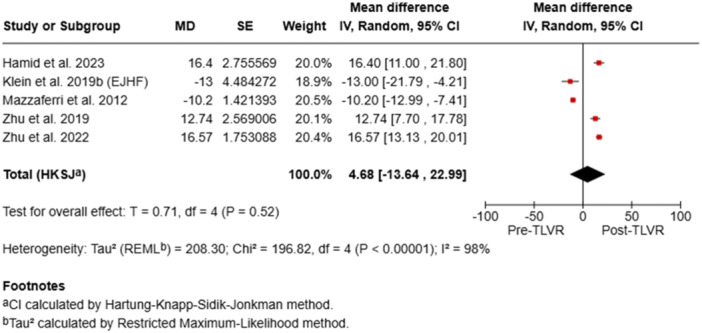
Pooled analysis of studies. [Color figure can be viewed at wileyonlinelibrary.com]

**Figure 5:**
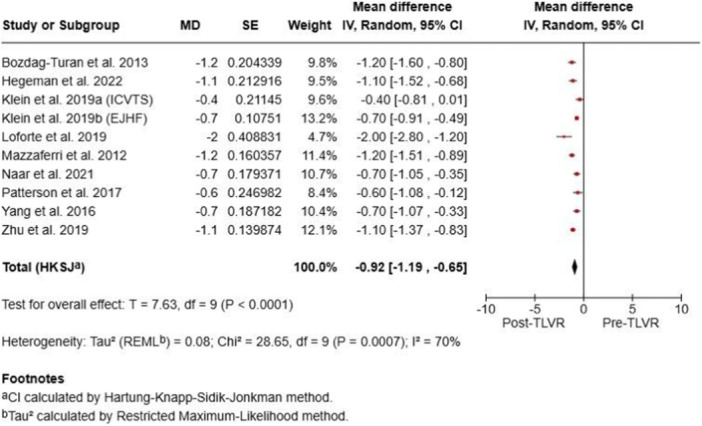
Pooled analysis of studies. [Color figure can be viewed at wileyonlinelibrary.com]

To minimize repetition, forest plots of pooled outcomes (LVEF, LVEDV, and NYHA class) were generated (Figures 2, 3, 4, 5), providing concise visualization of consistent post‐TLVR improvements across studies.

## 4 Discussion

To our knowledge, this is the first systematic review to evaluate the efficacy and safety of various transcatheter left ventricular restoration (TLVR) using the Revivent TC, Parachute HeartTech LVPD, or AccuCinch devices.

TLVR is designed to reduce LV volumes and restore physiologic geometry in patients with ischemic cardiomyopathy [4]. While huge research has been conducted on surgical reconstruction of the left ventricle, evidence remains variable, and the procedure is usually reserved for selected patients at centers of excellence [5]. The STICH trial, which compared CABG alone to CABG plus surgical LV aneurysmectomy, failed to demonstrate a survival benefit [6].

In contrast, TLVR represents a percutaneous alternative to surgical ventricular reconstruction (SVR), such as that studied in the STICH and RESTORE trials. Unlike SVR, which requires cardiopulmonary bypass and ventriculotomy, TLVR aims to achieve comparable geometric restoration and reduction in wall stress through a catheter‐based approach, potentially lowering procedural morbidity and recovery time.

TLVR was seen as a minimally invasive choice, particularly for patients with anteroapical aneurysm or akinetic scar after myocardial infarction, who might benefit from reducing the volume of the left ventricle [5]. Early outcomes from studies investigating TLVR show encouraging gains [7]. Klein et al recently published substantial decreases in LV volumes, improvement in ejection fraction, and symptomatic benefit at 12 months using the Revivent anchoring system. In this study, patients with a lower LVESVI demonstrated the greatest response [8]. Similarly, Wang et al recently showed significant benefits with Revivent TC in terms of LV volume, ejection fraction, 6‐min walk test and NYHA heart failure class, 9 months after the operation [9].

### 4.1 Comparison With Prior Studies

Our meta‐analysis reinforces these observations seen in other studies and show consistent benefit with TLVR throughout the trials. While baseline LV volumes were heterogeneous between the studies, TLVR was uniformly linked with significant reverse remodeling of the ventricle and functional improvement. Our meta‐analysis showed a mean reduction of LVEDV of –25.94 mL (95% CI: –33.66 to –18.22; *p* < 0.00001) and improvement of LVEF by +6.69% (95% CI: 4.68–8.70; *p* < 0.00001). Functional outcomes mirrored these structural gains, with mean NYHA class reduction of –0.73 (95% CI: –1.29 to –0.18; *p* = 0.02). Quality of life, as defined by KCCQ, was slightly better but not significant (MD + 4.68; 95% CI: –13.64 to 22.99; *p* = 0.52).

### 4.2 Heterogeneity and Patient Selection

Heterogeneity among studies likely reflects differences in device design (e.g., apical vs. basal remodeling mechanisms), patient selection (extent and location of scar, baseline LV size), and procedural learning curve. For instance, apically applied systems such as the Parachute device may favor patients with anteroapical dyskinesis, whereas Revivent and AccuCinch systems address broader LV geometry and wall stress reduction.

All devices reduced LVEDV and improved LVEF, with Revivent and AccuCinch showing the greatest benefit. Parachute showed the greatest variability amongst studies with excellent results seen in some and slight benefit seen in others. This variability underscores the importance of patient‐device matching and highlights a key source of heterogeneity in reported outcomes. Variability across studies can be attributed to heterogeneity in patient populations, differences in device deployment techniques, and inclusion of patients unlikely to derive benefit from TLVR.

### 4.3 Clinical Implications

The observed improvements in LV geometry and function suggest that TLVR may serve as an adjunctive or bridging option in the heart failure management algorithm—potentially fitting between guideline‐directed medical therapy (GDMT) and advanced interventions such as cardiac resynchronization therapy (CRT) or left ventricular assist device (LVAD) implantation. By targeting structural remodeling rather than solely hemodynamic optimization, TLVR offers a mechanistic complement to existing therapies.

In current heart failure management, transcatheter left ventricular restoration (TLVR) represents a minimally invasive option for patients with ischemic cardiomyopathy who remain symptomatic despite guideline‐directed therapy and are not candidates for surgical reconstruction or durable LVAD implantation. By restoring ventricular geometry and reducing end‐diastolic volume, TLVR can enhance systolic function and functional capacity while avoiding the morbidity of open surgery or long‐term mechanical support. As evidence and experience expand, TLVR may assume an intermediate role within existing heart failure algorithms, complementing CRT and bridging the gap between medical therapy and advanced mechanical interventions (4, 8, 9). Future heart failure guidelines may consider TLVR as a bridge or adjunct to CRT or LVAD in carefully selected patients with ischemic cardiomyopathy.

### 4.4 Limitations

Our findings in the meta‐analysis are limited by heterogeneity of study design, small sample size, and heterogeneous follow‐up. Results long‐term, particularly regarding survival and rates of hospitalization, are understudied.

Publication bias may also be present given the predominance of early feasibility and single‐arm studies. Additionally, the lack of randomized controlled trials and absence of standardized endpoints restrict generalizability. Long‐term data regarding mortality, rehospitalization, and device durability remain limited.

High statistical heterogeneity (I² > 90%) reflects variability in patient characteristics, device mechanisms, and procedural experience, warranting cautious interpretation. Finally, selective reporting and absence of unpublished negative data could have further influenced pooled estimates.

## 5 Conclusion

In conclusion, TLVR is emerging as a promising, minimally invasive therapy for ischemic cardiomyopathy, consistently achieving meaningful reverse remodeling and symptomatic improvement throughout all studies. Large multicenter randomized studies need to determine the long‐term durability of these effects and improve patient selection criteria for these devices. Future work should explore its integration within multidisciplinary heart failure management pathways, determine long‐term survival and hospitalization impacts, and refine patient selection based on anatomical and functional criteria.

## Conflicts of Interest

The authors declare no conflicts of interest.

## Supporting information


**Figure 1 Supplementary Table:** Quality Assessment Using Newcastle‐Ottawa Scale (NOS).
