# Supplementary material for: Transcatheter Left Ventricular Restoration in Ischemic Heart Failure and Dilated Cardiomyopathy
Source: Catheter Cardiovasc Interv. 2025 Dec 15;107(4):898–908. doi: 10.1002/ccd.70425 (PMC12953211; doi:10.1002/ccd.70425)
Supplement: Supplementary file 1 — Figure 1 Supplementary Table: Quality Assessment Using Newcastle‐Ottawa Scale (NOS). [file CCD-107-898-s001.docx]

Figure 1 Supplementary Table: Quality Assessment Using Newcastle-Ottawa Scale (NOS)

| Study | Year | Selection | | | | Comparability | | Exposure | | |  |
| --- | --- | --- | --- | --- | --- | --- | --- | --- | --- | --- | --- |
|  |  | Adequate case definition | Representativeness of cases | Selection of controls | Definition of controls | Study controls for age | Study controls for additional factor (sex, Edu, SE, dis.) | Ascertainment of exposure | Same method of ascertainment for cases and controls | Non-response rate | Score |
| Alaiti et al. | 2016 | ★ | ★ | ✖ | ✖ | ✖ | ✖ | ★ | ★ | - | 4 |
| Bozdag-Turan et al. | 2013 | ★ | - | ✖ | ✖ | ✖ | ✖ | ★ | ★ | - | 3 |
| Costa et al. (Parachute FIH) | 2012 | ★ | ★ | ✖ | ✖ | ✖ | ✖ | ★ | ★ | - | 3 |
| Costa et al. | 2014 | ★ | ★ | ✖ | ✖ | ✖ | ✖ | ★ | ★ | - | 3 |
| Hamid et al. | 2023 | ★ | ★ | ✖ | ✖ | ✖ | ✖ | ★ | ★ | ★ | 5 |
| Hegeman et al. | 2022 | ★ | ★ | ✖ | ✖ | ✖ | ✖ | ★ | ★ | ★ | 5 |
| Klein et al. (ICVTS) | 2019 | ★ | - | ✖ | ✖ | ✖ | ✖ | ★ | ★ | - | 3 |
| Klein et al. (Revivent TC) | 2019 | ★ | ★ | ✖ | ✖ | ✖ | ✖ | ★ | ★ | ★ | 5 |
| Loforte et al. | 2019 | ★ | - | ✖ | ✖ | ✖ | ✖ | ★ | ★ | - | 3 |
| Mazzaferri et al. | 2012 | ★ | ★ | ✖ | ✖ | ✖ | ✖ | ★ | ★ | - | 4 |
| Naar et al. | 2021 | ★ | ★ | ✖ | ✖ | ✖ | ✖ | ★ | ★ | ★ | 5 |
| Thomas et al. (PARACHUTE III) | 2015 | ★ | ★ | ✖ | ✖ | ✖ | ✖ | ★ | ★ | ★ | 5 |
| Patterson et al. | 2015 | ★ | - | ✖ | ✖ | ✖ | ✖ | ★ | ★ | - | 3 |
| Yang et al. | 2016 | ★ | ★ | ✖ | ✖ | ✖ | ✖ | ★ | ★ | - | 4 |
| Yun et al. | 2017 | ★ | - | ✖ | ✖ | ✖ | ✖ | ★ | ★ | - | 3 |
| Zhu et al. | 2019 | ★ | ★ | ✖ | ✖ | ✖ | ✖ | ★ | ★ | - | 4 |
| Zhu et al. | 2022 | ★ | ★ | ✖ | ✖ | ✖ | ✖ | ★ | ★ | - | 4 |
